# Panmictic and Clonal Evolution on a Single Patchy Resource Produces Polymorphic Foraging Guilds

**DOI:** 10.1371/journal.pone.0133732

**Published:** 2015-08-14

**Authors:** Wayne M. Getz, Richard Salter, Andrew J. Lyons, Nicolas Sippl-Swezey

**Affiliations:** 1 Dept. ESPM, UC Berkeley, CA 94720-3114, United States of America; 2 School of Mathematical Sciences, University of KwaZulu-Natal, Private Bag X54001, Durban 4000, South Africa; 3 Computer Science Dept., Oberlin College, Oberlin, Ohio, OH 44074, United States of America; 4 Vice Provost Office for Undergraduate Education, Stanford University, Stanford, CA 94305, United States of America; 5 Francis I. Proctor Foundation for Research in Ophthalmology, UC San Francisco, Box 0412, San Francisco, CA 94143-0412, United States of America; Tel Aviv University, ISRAEL

## Abstract

We develop a stochastic, agent-based model to study how genetic traits and experiential changes in the state of agents and available resources influence individuals’ foraging and movement behaviors. These behaviors are manifest as decisions on when to stay and exploit a current resource patch or move to a particular neighboring patch, based on information of the resource qualities of the patches and the anticipated level of intraspecific competition within patches. We use a genetic algorithm approach and an individual’s biomass as a fitness surrogate to explore the foraging strategy diversity of evolving guilds under clonal versus hermaphroditic sexual reproduction. We first present the resource exploitation processes, movement on cellular arrays, and genetic algorithm components of the model. We then discuss their implementation on the Nova software platform. This platform seamlessly combines the dynamical systems modeling of consumer-resource interactions with agent-based modeling of individuals moving over a landscapes, using an architecture that lays transparent the following four hierarchical simulation levels: 1.) within-patch consumer-resource dynamics, 2.) within-generation movement and competition mitigation processes, 3.) across-generation evolutionary processes, and 4.) multiple runs to generate the statistics needed for comparative analyses. The focus of our analysis is on the question of how the biomass production efficiency and the diversity of guilds of foraging strategy types, exploiting resources over a patchy landscape, evolve under clonal versus random hermaphroditic sexual reproduction. Our results indicate greater biomass production efficiency under clonal reproduction only at higher population densities, and demonstrate that polymorphisms evolve and are maintained under random mating systems. The latter result questions the notion that some type of associative mating structure is needed to maintain genetic polymorphisms among individuals exploiting a common patchy resource on an otherwise spatially homogeneous landscape.

## Introduction

Computational modeling of population interactions is a growing research endeavor [1] in the context of including behavioral, physiological or genetic heterogeneity among individuals living in spatiotemporally heterogenous stochastic environments [2]. This endeavor is facilitated by ever more powerful computational tools, both hardware (easier and more cost effective access to distributed computing resources) and software (more accessible and user friendly software interfaces for model prototyping, development and deployment) that overcome the limits of analytical and dynamical systems modeling frameworks for addressing both theoretical and applied problems. Access to computational technologies requires either strong coding fluency and skills, or modeling platforms that minimize the need for such fluency and skills.

The last decade has seen considerable progress in the development of user-friendly modeling software. Numerous platforms such as Vensim [3], Stella [3], Simile [3, 4], Insight Maker [5] and Berkeley Madonna [6] now provide flow-chart-like visual environments for modeling dynamical systems. Other platforms, such as NetLogo [7], Simile [4], Insight Maker [5], Repast Simphony [8] and AnyLogic [9] facilitate agent-based modeling (ABM). These platforms, with varying degrees of success, meet the following principles that have guided the development of the Nova platform [10, 11] used in the simulations presented here. Platforms should: 1.) be accessible to students with noncoding skills when it comes to developing didactic models that have either or both dynamical systems and ABM capabilities; 2.) emphasize model design principles by accessibly organizing and coordinating the details of model implementation; 3.) incorporate a versatile, “Turing complete” (i.e., computationally universal) language that facilitates model sharing online (e.g. as web applications); 4.) be powerful enough to build models able to address cutting-edge research questions in the basic and applied sciences.

In this paper, we discuss the characteristics of the Nova platform in the context of principles 1–4 articulated above, through the application of genetic (sometimes referred to as evolutionary) algorithms to addressing questions in population biology. Genetic algorithms have been applied to ecological problems for at least three decades, although it was not referred to as such or in terms of its alternative evolutionary algorithm designation, in its application by Poethke and Kaiser in 1985 to the evolution of time-sharing behavior in a Dragonfly mating system [12]. In the early 1990’s, Stockwell, Nobel and colleagues developed machine-learning methods, in particular a framework called GARP (Genetic Algorithms for Ruleset Production) [13], which since then has been frequently used to study species distributions [14–16]. Genetic algorithms have also been used *inter alia* to study the evolution of female preference as it relates to male age [17], dispersal in insects [18] including the potential impact of climate change on the geographical distribution of the Argentine ants [19], insect-plant [20], and prey-predator studies [21], as well as ecotoxicology [22], landuse change [23] and other challenging questions [24].

A discussion of genetic algorithms is included in a review by Olden et al. [25], while Holzkämper et al. [23] provide an informative diagram on the implementation of genetic algorithms in their study on managing landscapes to enhance species diversity. Here, for the sake of completeness, we provide a broad overview of GA terms and methods, focusing on the application of GAs to models of agents foraging on a patchy landscape. Specifically, following the lead of earlier work on the application of artificial intelligence methods to movement-related decision processes of animals in heterogeneous environments [26], we first provide details of the within-patch, consumer-resource interaction components and movement decisions components of our model, and then provide details of the GA component of our model, particularly as it relates to mutational and reproductive processes. Subsequently, we examine hypotheses regarding the evolution of movement-type polymorphisms in populations of individuals foraging on patchy landscapes and reproducing clonally versus hermaphroditically. Our model includes the ecological processes of competition among individuals and the dynamical response of exploited resources. The total biomass production over all individuals is recorded at the end of each generation, but only the most successful individuals (i.e. those experiencing the greatest increase in biomass), however, are involved in reproduction.

## Methods

### Simulation framework

**Fig 1:**
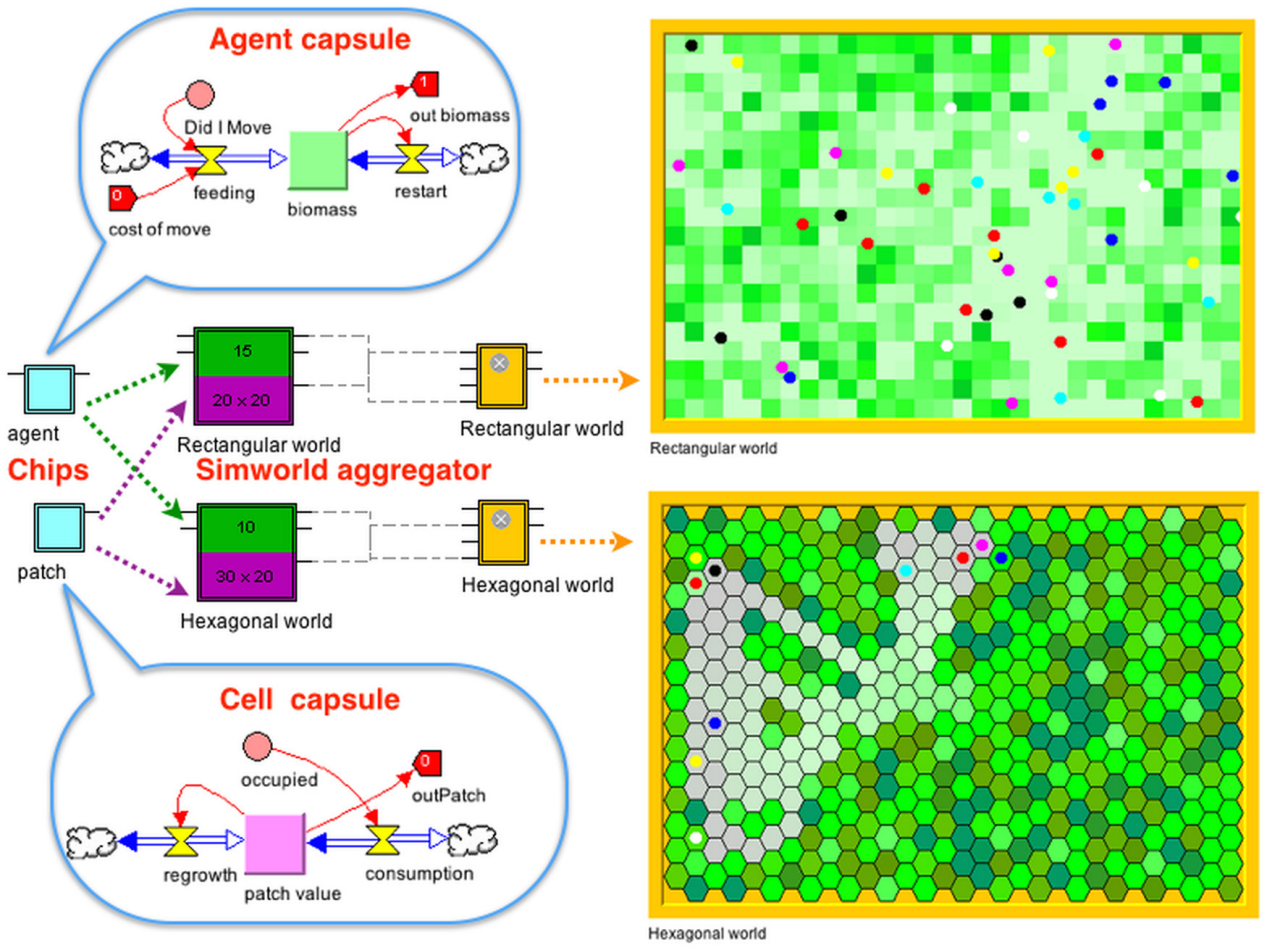
Stock-and-flow icons are used to graphically build systems of difference (lavender-pink square in cell capsule) and differential (lime-green square in agent capsule) equations that Nova encapsulates as *chips with input output pins* (pale blue squares) at higher levels of representation, through drag and drop construction. Agent and cell capsules respectively dropped onto the clover and purple colored components of an *aggregator* that creates an array of agents able to move over either a *rectangular or hexagonal cellular array*, of specifiable dimensions with toroidal or non-toroidal topologies.

**Fig 2:**
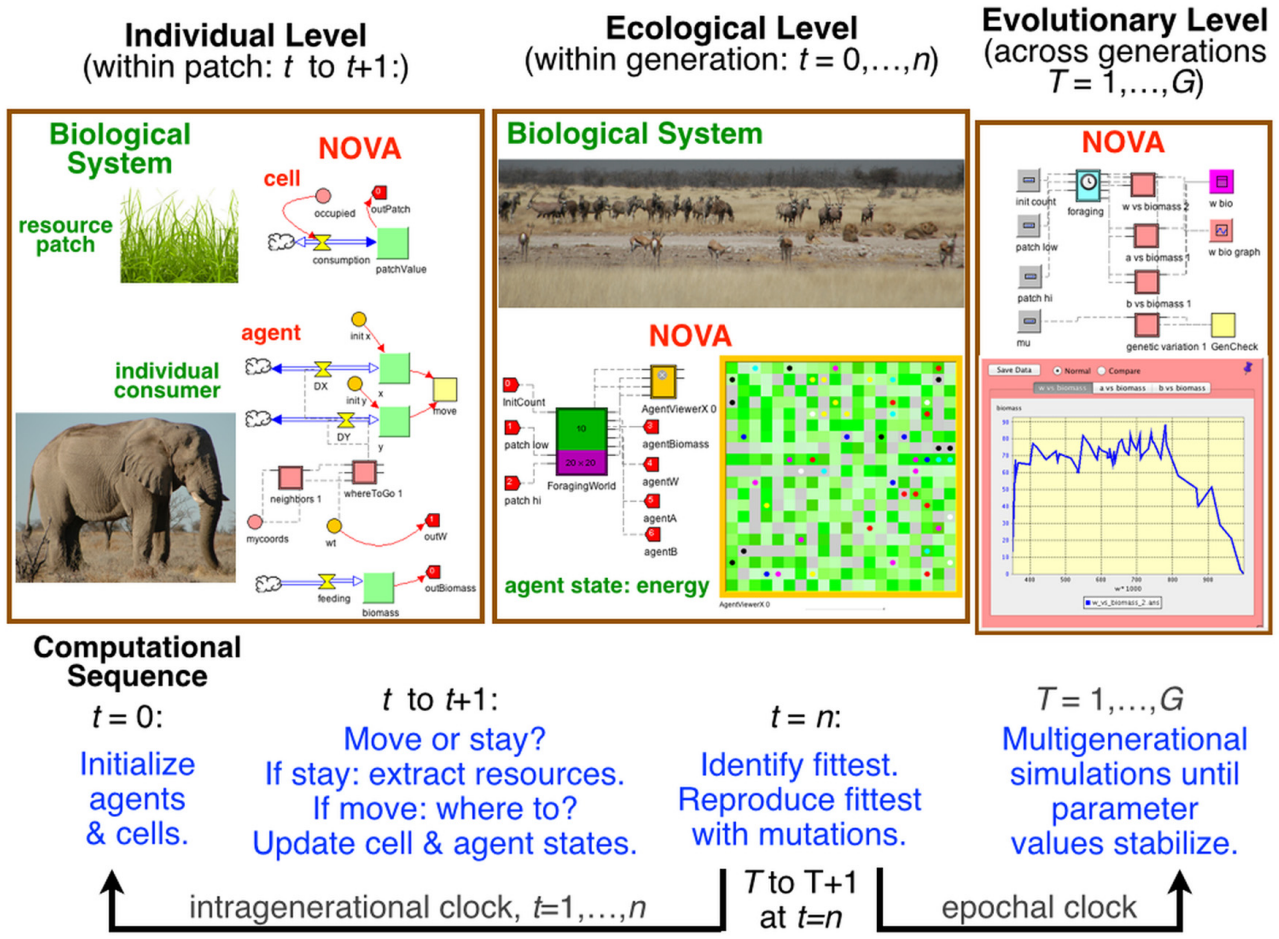
Left hand panel: *individual level* processes controlling the replenishment of resource patches and the growth (biomass) of individuals, through extraction of resources from these patches, are depicted using graphical Nova elements. Central panel: *ecological level* process managed by a Nova *simworld aggregator* are graphically depicted, with consumers either foraging within patches or moving from patch-to-patch during each tick of the intragenerational clock. The progeny of the fittest individuals (i.e., greatest biomass at *t* = *n*) inherit perturbed (mutational process) parameter values from one (clonal reproduction) or two (sexual reproduction) parents at the end of each generation. Right panel: the *evolutionary level* process, represented by changes to the parameter values of individuals (i.e., genotypes and corresponding phenotypes), is monitored by the *epochal clock* that runs the evolutionary algorithm for *G* generations. (See S1 Text for discussion of Nova platform).

Our simulation model was built using the Nova platform [10], which is highly modularized in terms of: 1.) first creating dynamic agent “capsules” that interact with their environments through input/output interfaces and 2.) the allowing these agents to move over a heterogeneous resource landscape. Movement can either be on a Cartesian plane or from one cell to another on a rectangular or hexagonal array of specifiable dimension (Fig 1; see S1 Text for more details of the platform). Our model moves agents over rectangular arrays of variable and dynamic resource cells and was designed to address questions regarding the efficiency of behavioral guilds of foragers. The model itself operates at three time scales: i) a within patch foraging scale (one tick of the intragenerational clock); ii) an intragenerational patch-to-patch movement scale (*n* ticks of the intrageneration clock equal to 1 tick of the intergenerational clock); and iii) an intergenerational evolutionary scale (*G* ticks of the epochal clock), as depicted in Fig 2. At the within-patch and intragenerational scales, a cohort of individuals of specified size is followed. During their lifespan, these individuals (i.e. agents) live on a cellular-array landscape where each cell has a single resource value. The agents consume resources at their current location, gain biomass (which serves as a proxy for evolutionary fitness), and make decisions about movement, based on their local resource environment and locations of neighboring competitors (see details below). Landscape cells grow in resource value when not consumed. At the end of each cohort’s pre-determined lifespan, intergenerational reproductive and evolutionary processes are simulated. The biomass of each individual is tallied as a proxy for fitness, and the most fit individuals (top half of the cohort) are allowed to reproduce. In our simulations involving clonal reproduction, a mutational process was included by allowing parameter values of progeny to be stochastic perturbed from those of their parent, using an approach elaborated below. In an initial set of ‘quick and dirty’ sexual reproduction simulations used to generate hypotheses, we allowed the fittest half of the population to choose partners at random and then set the parameter values of their progeny to be the average of the parameter values of the parents involved, subject to mutations of these values. In a subsequent set of sexual reproduction simulations, we made the process more realistic by incorporating diploid genetic structure, random segregation and codominant phenotypic expression of alleles. Once the new generation of individuals (progeny) was created in all three approaches to reproduction, we then assigned an initial location on the landscape at random, and the intragenerational simulation cycle began again. This cycle continued for a pre-determined number of generations, as indicated in Fig 2.

We first carried out a set of exploratory simulations, using parameters specified in S1 Table, with the interpretations of these parameters provided for clarity. Based on these results, we then formulated the hypothesis that movement behavioral guilds of foragers evolve to collectively be more efficient in exploiting resources (in the context of our model) under clonal than under random hermaphroditic reproduction. To explore this question, we modified the sexual reproduction process within our model to be a diploid co-dominant process in which the genotype of each individual’s parameter values (three parameters in our case) are each specified by two alleles. These alleles were subjected to mutations during each reproductive cycle, with each individual’s parameter phenotype being the average of the two allelic values. The results of this study are reported in the section following the presentation of the exploratory simulation results.

### Genetic algorithms from a biological perspective

In the computer science field of artificial intelligence, a genetic algorithm (GA) is a search heuristic that mimics the process of natural selection [24]. In modeling in the applied sciences, a GA is a numerical method, mimicking evolution, to select agents (models) with sets of parameter values that optimize some measure associated with the performance of the agents [25]. For example, in the context developed here, the model deals with the efficiency of individual consumers in exploiting a landscape of patchily distributed resources. Much of the jargon, but not all, associated with GAs comes from evolutionary theory: some of it is summarized in Table 1.

**Table 1:** Terms and definitions used in genetic algorithms (GAs).

Term	Definition
Phenotype	an agent of a particular behavioral, physiological, or morphological type
Fitness function	a measure associated with the agent that will be optimized
Genome	a set of parameter values associated with an agent
Genotype	a specific genome
Mutation	a perturbation of a parameter value in the genome
Simulated annealing*	mutational perturbations decrease in size over time
Cloning reproduction	duplication of a genotype (with mutations)
Sexual reproduction	genotype values generated from two parents (with mutations)
Codominant	phenotype is average of parental genotype for gene concerned
Hard selection	best group of individuals produce a fixed number of progeny
Soft selection	better than worse individuals more likely to reproduce
Generation time	the model run time over which fitness is assessed
Epoch	the number of generations over which the evolutionary process is assessed

*see Eq 1

The seminal text on genetic algorithms (GA), formulated within a general adaptive systems framework, is Holland’s 1975 book: *Adaptation in Natural and Artificial Systems* (1992 Edition: [27]). More recently, the application of GAs is widely spread within the computer science and artificial intelligence communities, but has only occasionally been applied to economic systems [28], reviewed for a general audience from a bit-string coding point of view [29], reviewed for a chemistry audience [30], applied to evolutionary questions in ecology [20, 31, 32], used to assess the design of sensory systems in animals [33], and to asses the efficiency of decision processes in movement ecology [34, 35].

The essential features of a genetic algorithm is that it consists of at least two temporal frames (Fig 2): an intragenerational frame in which individuals are governed by processes that affect their life-time fitness; and an intergenerational frame in which generations of individuals succeed one another, with the fittest individuals in each generation being the most likely to pass on the traits promoting that fitness to future generations. The intragenerational frame itself may be further refined so that the processes determining the life-time fitness of individuals can be dynamically modeled over the life time of individuals. This integration across time scales is depicted in Fig 2 in the context of consumer-resource interactions, where our surrogate for a measure of fitness is individual biomass, as determined by growth processes dependent on resource extraction over the life time of individual consumers.

The genetic algorithm begins by initializing the system *t* = 0 (start of the intragenerational clock) and *T* = 1 (the first generation). In our exemplar, the initial state of agent *A*_*a*_, *a* = 1, …, *N*_*A*_, is its starting biomass *B*_*a*_(0), and is located at *L*_*a*_(0) = *C*_*i*,*j*_, where *C*_*i*,*j*_ are *cells* on a rasterized two-dimensional landscape or *cellular array* (row *i* and column *j*). The initial state of each cell is *R*_*i*,*j*_(0) at the start of each intragenerational cycle. In moving the intragenerational clock forward from *t* to *t* + 1, *t* = 0, …, *n*, we query each agent as to whether it will move or stay to exploit the resources in its current cell. If it does move, we then determine to which cell it moves. The outcome of these calculations for agent *A*_*a*_ is represented by the value of its *movement designator* $M_{{\text{p}}_{a}}^{a}$, as discussed in the next section, where **p_a_** is a set of agent-specific parameter values. After computation, the state *B*_*a*_(*t*) and location *L*_*a*_(*t*) of agent *A*_*a*_, and the state *R*_*i*,*j*_(*t*) of all cells *C*_*i*,*j*_ are updated, as elaborated In the intergenerational updating subsection below.

Once the updating process is completed, we continue for *t* = 1, …, *n* − 1, ultimately reaching a final state *B*_*a*_(*n*) for each of the agents *A*_*a*_. The agents *A*_*a*_ are then sorted according to their final state values *B*_*a*_(*n*) from, say, largest to smallest. A number of different reproductive systems are possible including clonal (apomictic or automictic) versus sexual reproduction (hermaphroditic or distinct sexes, assortative or disassortative mating or different levels of inbreeding), as are patterns of inheritance (e.g. using co-dominant versus dominant-recessive relationships) and processes relating to linkage and genetic crossover. Most of our exploratory simulations use the following clonal reproduction process.

The *N*_*A*_ agents simulated over each generation are ranked by biomass value *B*_*a*_(*n*) (the fitness measure) and the fittest half are selected to reproduce. Each of the selected agents is cloned by creating a copy with the same parameter set **p_a_**. Each of the cloned values within **p_a_** are then individually perturbed by differing amounts, each amount drawn at random on the interval [−*μ*, *μ*] for some suitably small value *μ* > 0. The fittest half of the current generation and their mutated clones constitute *N*_*A*_ individuals used to start the next generation simulation. The maximum allowable perturbation *μ* to the values in **p_a_** decreases as the generations progress over *T* = 1, …, *G* (i.e. simulated annealing) so that *μ* is regarded as a decreasing function of *T*—i.e., *μ* ≡ *μ*(*T*), where $\frac{d\mu }{dT}\leq 0$: this allows for initial rapid progress towards the emergence of the fittest individuals, followed by smoother convergence to the fittest group of individuals. We note that further investigations are needed to evaluate how likely it is that solutions are local or global maxima. In our simulations, for perturbation parameter values *μ*_0_ > > *μ*_∞_ > 0 and time parameter values *ψ* > 0, we expressed *μ*(*T*) as

$$\mu \left(T\right)=\frac{\left(\mu_{0}-\mu_{\infty }\right)}{1+{\left(T/\psi \right)}^{3}}+\mu_{\infty }$$ (1)

which satisfies *μ*(0) = *μ*_0_, *μ*(*ψ*) = (*μ*_0_ − *μ*_∞_)/2 and lim_*T* → ∞_ *μ*(*T*) = *μ*_∞_. The intergeneration computations are then repeated for *T* = 1,2,…, until terminating at *T* = *G*, the end of the Epoch, at which point the genotypes (parameter values) are noted and the performance of the phenotypes evaluated.

The exploratory simulations were then followed by a comparison of clonal with random hermaphroditic sexual mating, and under an assumption of diploid genetics under codominance for the three trait parameters involved. Thus, for the diploid system, the progeny parameter values are averages of two alleles: one from each parent segregated at random during “meiosis.”

### Landscape perception and movement computations

#### General Movement Process Algorithm

The movement process itself, as described in general terms in Nathan et al. [36], requires an agent to make movement decisions based both on its internal state and the state of its environment. The sequence of information considered as part of the general process of making the decision *where and when to move*, is follows.

Evaluate environment: each agent *A*_*a*_, *a* = 1, ⋯, *N*_*A*_, computes a state vector **E**_*a*_(*t*) associated with its local environment, as it may pertain to the location of
  - resources such as food, water or shelter
  - conspecifics for either protection, social or antagonistic interactions, mitigation of competition, fear, or mating behavior
  - location of heterospecifics that may be competitors, predators, prey items, etc.
Initiate movement decision computation: each agent feeds the environmental information computed above, along with its internal state—such as, degree of hunger, thirst, fear, states of internal diurnal and seasonal clocks, memory states—into its “brain.”
Complete movement decision computation: based on environmental input **E**_*a*_(*t*) and internal state **B**_*a*_(*t*), agent *A*_*a*_ carries out a computation that yields a movement designator $M_{{\text{p}}_{a}}^{a}\left({\text{E}}_{a}(t),{\text{B}}_{a}(t))\right).$
Execute action: The designator $M_{{\text{p}}_{a}}^{a}\left({\text{E}}_{a}(t),{\text{B}}_{a}(t))\right)$ will either specify that agent *a* should stay (i.e., *L*_*a*_(*t* + 1) = *L*_*a*_(*t*)) or move to new location *L*_*a*_(*t* + 1) (identified as a specific cell *L*_*a*_(*t* + 1) = *C*_*i*,*j*_).

The ability of individuals to quantify their local environment will depend on the sensory machinery they possess. For example, they may have visual capabilities with acuity reduced with distance and influenced by the local topography of the landscape; or, they may be able to process olfactory or auditory cues that depend on local wind direction and landscape features. This information, along with information on their current internal states, is then fed into a computational module that uses either a mathematical function or a dynamical systems computation to produce an answer to the questions being addressed.

As already mentioned, in the Nova platform agents can move over a landscape specified by a rectangular or hexagonal cellular array of any dimension. The two approaches are contrasted in S2 Text, though we use the Moore Neighborhood approach outlined below, where movement is formulated in terms of eight neighboring cells that occur on rectangular arrays (cf. Figure in S2 Text).

#### Moore Neighborhood Movement (resources and competitors)

The three-parameter model, presented here, is but one example of how to set up a movement decision process that considers tradeoffs among the relative values of obtaining resources, avoiding competition, and investing energy to move.

1. For all cells in the array, using a single index label *k* (e.g. *C*_*i*,*j*_, *i* = 1, …, *n*_*i*_, *j* = 1, …, *n*_*j*_ can be labeled *k* = *i* + (*j* − 1)*n*_*i*_, for *k* = 1, …, *n*_*i*_ *n*_*j*_), *R*_*k*_ denotes the resources in cell *k* and $R_{k}^{M}$ denotes the average of the resources across all eight cells in the Moore neighborhood of cell *k*. Note that at an individual agent level a local neighbor labeling is more convenient (cf. left panel of Figure in S2 Text), in which case later translation to a global labeling of cells is required.
2. Similarly, for all cells in the array, *J*_*k*_ denotes the number of agents in cell *k* and $J_{k}^{M}$ denotes the average number of agents in the eight cells of the Moore neighborhood of cell *k*.
3. For each agent *A*_*a*_, *a* = 1, …, *N*_*A*_, at time *t*, identify its current location *L*_*a*_(*t*).
4. Then labeling *L*_*a*_(*t*) as cell 0, for agent generate the environmental vector introduced in the previous subsection.
  $$E_{a}\left(t\right)=\left\{R_{0},J_{0},R_{1},R_{1}^{M},J_{1},J_{1}^{M},\ldots ,R_{8},R_{8}^{M},J_{8},J_{8}^{M}\right\},$$
  which is then used to implement (in a series of logical statements) the movement designator $M_{{\text{p}}_{a}}^{a}$.
5. Given parameters *α* ≥ 0 and *δ* ≥ 0, which we will respectively refer to as the *neighbor-discount* and *competition-tradeoff* parameters, assign the following values to each of the eight cells neighboring *C*_*k*_:
  $$V_{\ell }=R_{\ell }-\delta J_{\ell }+\alpha (R_{\ell }^{M}-\delta J_{\ell }^{M})\;\ell =0,\ldots ,8$$
6. Given parameter *ρ* ≥ 0, which we refer to as the *movement-threshold* parameter, apply the movement rule
  $$\begin{array}{l} If\;\;V_{0}>0\text{ and}\;\frac{V_{l}}{V_{0}}<\rho \;i=1,\ldots ,6\;Then\;\;Stay\\ Else\;\text{Move}\text{ to cell}\;i,\;\;\text{where}\;\;i=\text{argmax}\{V_{l}|l=0,\ldots ,8\} \end{array}$$
  Note, in this algorithm, if the element *V*_0_ in the list is the largest, then the **Move** is to stay in the current cell, but the cost calculated in the updating section will be as though the individual moved. Of course, this can be modified in anyway we think appropriate. Also, note that the movement parameter vector **p**_*a*_ = (*α*, *δ*, *ρ*)′ is agent specific.

### Intragenerational updating

At the start of each generation, *T* = 1, …, *G*, the *simworld* aggregator (central green/purple chip in the middle panel in Fig 2) is initialized by assigning to each cell an initial resource value *R*_*k*_(0), *k* = 1, …, *n*_*i*_ *n*_*j*_ (i.e., using the single index convention mentioned in point 1 in the subsection above), that is chosen at random to be in the range [*R*_min_, *R*_max_]. Of course, more sophisticated approaches can assign the resource values in some aggregated or contagious way. The new group of agents *A*_*a*_, *a* = 1, …, *N*_*A*_, whose genotypes have been determined by the reproductive process outlined earlier, are then assigned to cells (the simplest is a random assignment), as well as assigned a set of initial state values *B*_*a*_(0), *a* = 1, …, *N*_*A*_, which in the simplest case may all be the same. Once these assignments have been made, the intragenerational ecological dynamics, in which cell resource values *R*_*k*_(*t*) and agent locations and values (*L*_*a*_(*t*), *B*_*a*_(*t*)) are updated, can be applied.

In formulating our updating equations, we confine ourselves to separately identifying the agent *A*_*a*_’s internal state *B*_*a*_(*t*) (i.e., its biomass) and location *L*_*a*_(*t*) at time *t* as scalar values. Also, in referring to the movement designator *M*_*a*_(*t*), we have dropped reference to the movement parameters **p**_*a*_; since, although they are agent specific, they are unchanging over each intragenerational simulation. For notational convenience, we use Agent_*k*_ to represent the set of *N*_*k*_(*t*) agents remaining in cell *C*_*k*_ after movement has been implemented at time *t*, and similarly use *N*_*a*_(*t*) to represent the number of agents occupying cell *L*_*a*_(*t*) at time *t*, after movement has occurred. We also consider the state of cell *k* only in terms of a scalar resource value *R*_*k*_(*t*), fully recognizing that a more general approach requires a vector description.

Since we are focusing on consumer-resource interactions [37–39], the key to specifying our updating equations at time *t* + 1 is first to evaluate the movement designators *M*_*a*_(*t*) for each agent, then to formulate the resources acquired by agent *A*_*a*_, if it stays within a cell to extract resources, and the resources extracted from cell *C*_*k*_ by those agents that do not move during the interval [*t*, *t* + 1]. With these conventions the following equations are used.

Intragenerational Updating Equations. After the initial agent state values *B*_*a*_(0), and agent cell locations *L*_*a*_(0) = *C*_*k*_ (the initial cell *k* that agent *a* is placed in), for some *k* = 1, …, *n*_*i*_ *n*_*j*_, have been selected for *a* + 1, …, *N*_*A*_, then for *t* = 1, …, *n* the following updating procedure is followed, employing the following parameters (which in more advanced applications can be made agent specific): the maximum resource extraction rate *u*, the extraction-efficiency parameter *h*, the competition parameter *q*, the biomass-conversion-rate parameter *κ*, the metabolic-loss-rate parameter *c*, the resource-intrinsic-growth-rate parameter *r*, reservoir parameter *g* and saturation parameter *s*:
  $$\begin{array}{ccc} \text{Calculate}\;L_{a}\left(t+1\right)\text{,}\;a=1,\ldots ,N_{A}, & & \text{using}\;\text{rules}\;\text{specified}\;\text{above}\\ \text{Identify}\;\text{agents}\;\text{remaining}\;\text{in}\;C_{k}\; & & \text{to}\;\text{obtain}\;\text{sets}\;\text{AGENT}{}_{k}\text{,}\;\text{size}\;N_{k}\left(t\right)\;\\ \text{Calculate}\;A_{a}\;\text{extraction}\;\text{in}\;C_{k}\text{:}\;F_{k}\left(t\right) & = & \min\{\frac{R_{k}\left(t\right)}{N_{k}\left(t\right)},\frac{uR_{k}\left(t\right)}{h+R_{k}\left(t\right)+qN_{k}\left(t\right)}\}\\ \text{If}\;\text{agent}\;A_{a}\;\text{Stays},\;\text{then:}\;B_{a}\left(t+1\right) & = & B_{a}\left(t\right)+\kappa F_{k}\left(t\right)\\ \text{If}\;\text{agent}\;A_{a}\;\text{Moves},\;\text{then:}\;B_{a}\left(t+1\right) & = & \left(1-c\right)B_{a}\left(t\right)\\ \text{Calc.}\;C_{k}\;\text{extraction}\;\text{by}\;\text{AGENT}{}_{k}\text{:}\;\;\;R_{k}^{*}\left(t\right) & = & R_{k}\left(t\right)-N_{k}\left(t\right)F_{k}\left(t\right)\\ \text{Update}\;C_{k}\text{:}\;R_{k}\left(t+1\right) & = & R_{k}^{*}\left(t\right)+r(1-\frac{R_{k}^{*}\left(t\right)}{s})(R_{k}^{*}\left(t\right)+g) \end{array}$$

We note that resource growth equation for *R*_*k*_(*t* + 1) is the same for each cell: that is, *r*, *s* and *g*, in the last equation above, are not cell specific. A thorough examination of the impacts of resource heterogeneity on the evolutionary ecology processes considered in this paper, requires these parameters to depend on *k*; and, also, *u*, *h* and *q* could be made patch specific. Considering this level of heterogeneity requires elaboration beyond the detail of this presentation.

### Epochal implementation

At the completion of each intergenerational cycle, after implementation of the reproductive process, the clock *T* is advanced, and the cycle is repeated (cf. the right-hand panel of Fig 2). During each reproductive cycle, the progeny of the fittest agents’ parameters are mutated, so it might be useful to monitor the reproducing-population average of this fitness measure (i.e. biomass of an agent at the end of its intragenerational cycle), as the population evolves.

It is useful during the simulation to keep track of the values of selected parameters to see how they evolve, since analyses of these values provide insight into the evolving movement decision tradeoffs in response to resource heterogeneity levels, intensity of competition, and other ecological parameters in the model. In our illustrative simulation results presented in the next section, we focus on the evolution of the average final biomass $\bar{B}(t)$ of the upper 50^*th*^ percentile of the agents (i.e., those that reproduce) in each generation. We also follow the evolving values of the *neighbor-discount* parameter *α*, the *competition-tradeoff* parameter *δ* and the *movement-threshold* parameter *ρ* in determining the movement decision making process of the fittest individuals, as the population evolves.

## Exploratory Analysis

A primary source of heterogeneity in our model is the initial resource value of each patch. Since this initial patch heterogeneity, compounded with stochastic aspects of the model, creates considerable variability in the output, we explored the behavior of the model with this heterogeneity removed: viz. we made the initial resource conditions near homogeneous by setting the baseline initial range of patch values to [2.99,3.00] (Table 2: note the 0.01 difference between the lower and upper values creates a small amount of stochasticity that renders initial movements of agents stochastic rather than deterministic). We carried out a number of runs where the initial resource patch values were random, uniformly distributed on [0,5.99] (i.e., the same mean as above); and found that all our results were qualitatively the same as those presented below.

**Table 2:** Baseline Parameter Values.

Parameter	Description	Value
[*R*_min_, *R*_max_]	patch initial resource range each generation	[2.99,3.00]
*s*	resource saturation in a patch	20
*g*	resource reservoir level	0.1
*r*	resource intrinsic growth rate	0.1
*u*	maximum extraction rate	10
*h*	half-saturation efficiency	20
*q*	intraspecific competition	0.5
*c*	cost of moving	0.1
*κ*	consumer biomass conversion	0.1
*μ*_0_	initial maximum mutation size*	0.1
*μ*_∞_	asymptotic maximum mutation size*	0.001
*ψ*	mutation-scaling*	50
*n*	length of intragenerational cycle	100
*G*	number of generations in epochal cycle	200
*α*	neighbor-discount	evolution
*δ*	competition-tradeoff	evolution
*ρ*	movement-threshold	evolution

*see Eq 1

In our first set of simulations, we considered the simplest case of two agents (*N*_*A*_ = 2) and executed three separate runs. The evolved parameter values and the average fitness values for the two agents at the final time *T* = 200 are provided in Table 3. The results suggest that the optimal *movement-threshold* value for parameter *ρ* is around the low 0.70s, while different combinations of values for the *neighbor-discount* and *competition-tradeoff* parameters *α* and *δ* respectively are possible. Some variation in fitness across runs occurs because the probability that two individuals get close to one another at any time during the simulation has a stochastic component, which then impacts individuals through competitive processes included in the resource extraction process and movement to patches previously visited by other consumers.

**Table 3:** Results for two agents.

Run	*α*	*δ*	*ρ*	Fitness Value
	competition tradeoff	neighbor discount	movement threshold	
1	0.73	0.14	0.75	32.6
2	0.33	0.22	0.76	32.8
3	0.46	0.54	0.72	33.1

**Fig 3:**
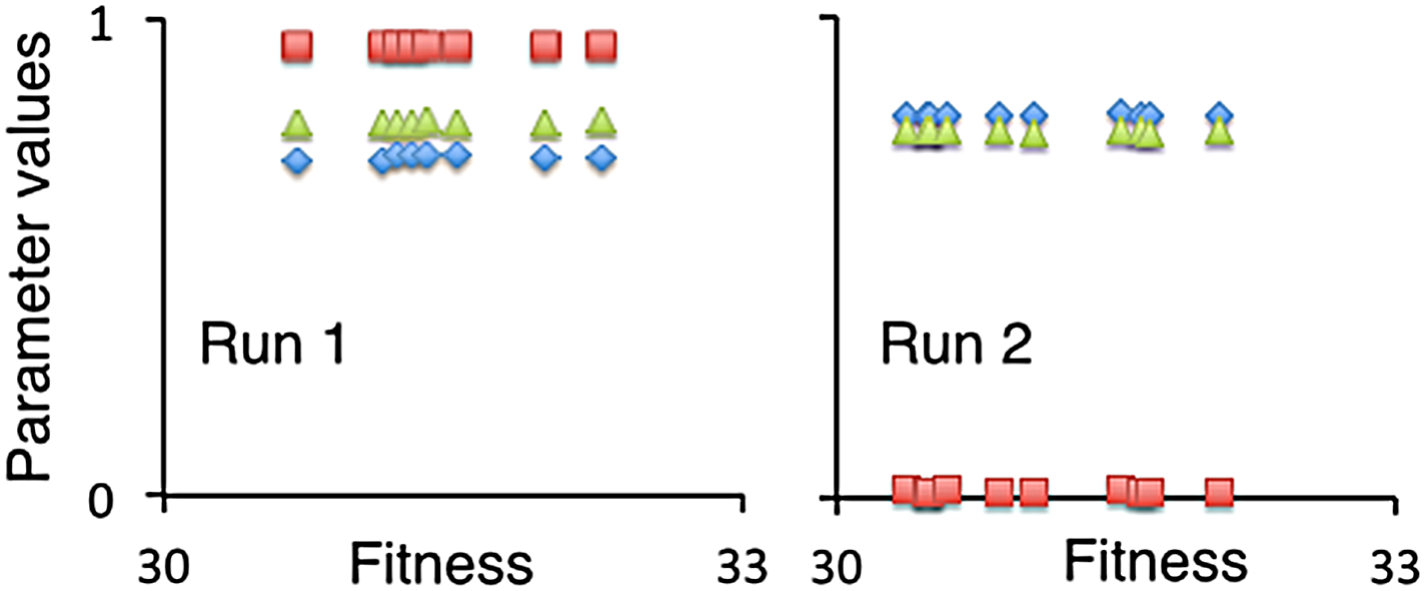
Evolved values of parameters (introduced in the Moore neighborhood movement section above). The left and right panels are the result from two different 10-agent runs using the same baseline data (Table 2). Parameter values for each of the ten agents in the final generation (*T* = 200) are plotted as a function of their fitnesses (ranging from 30 to 33). The evolved *neighbor-discount* parameter *α* (green triangles), *competition-tradeoff* parameter *δ* (red squares), and *movement-threshold* parameter *ρ* (blue diamonds), across the 10 agents differ, in the two runs; but yield similar fitness distributions, although the left panel shows a little more chance variance than the right.

In our second set of simulations, we considered the low-density case of ten agents (ten agents on 400 patches is a density of 0.025 individuals per patch). In two different runs, the population evolved to a particular movement type rather than to a coalition or guild of movement types (i.e., a movement-type monomorphism versus a polymorphism); but, again, the types differed from run to run, as illustrated in Fig 3 (note: fitness is on the horizontal axis in this figure but on the vertical axes in subsequent figures). In these two cases, the average fitness values were 31.0 and 28.5 respectively, which is around 6 to 14% less than in the two-agent case, the difference being explained by the stochastic variations in the cost of avoiding competition and the slightly lower availability of resources when individuals have 9 rather than just one other agent to avoid (as discussed below).

**Fig 4:**
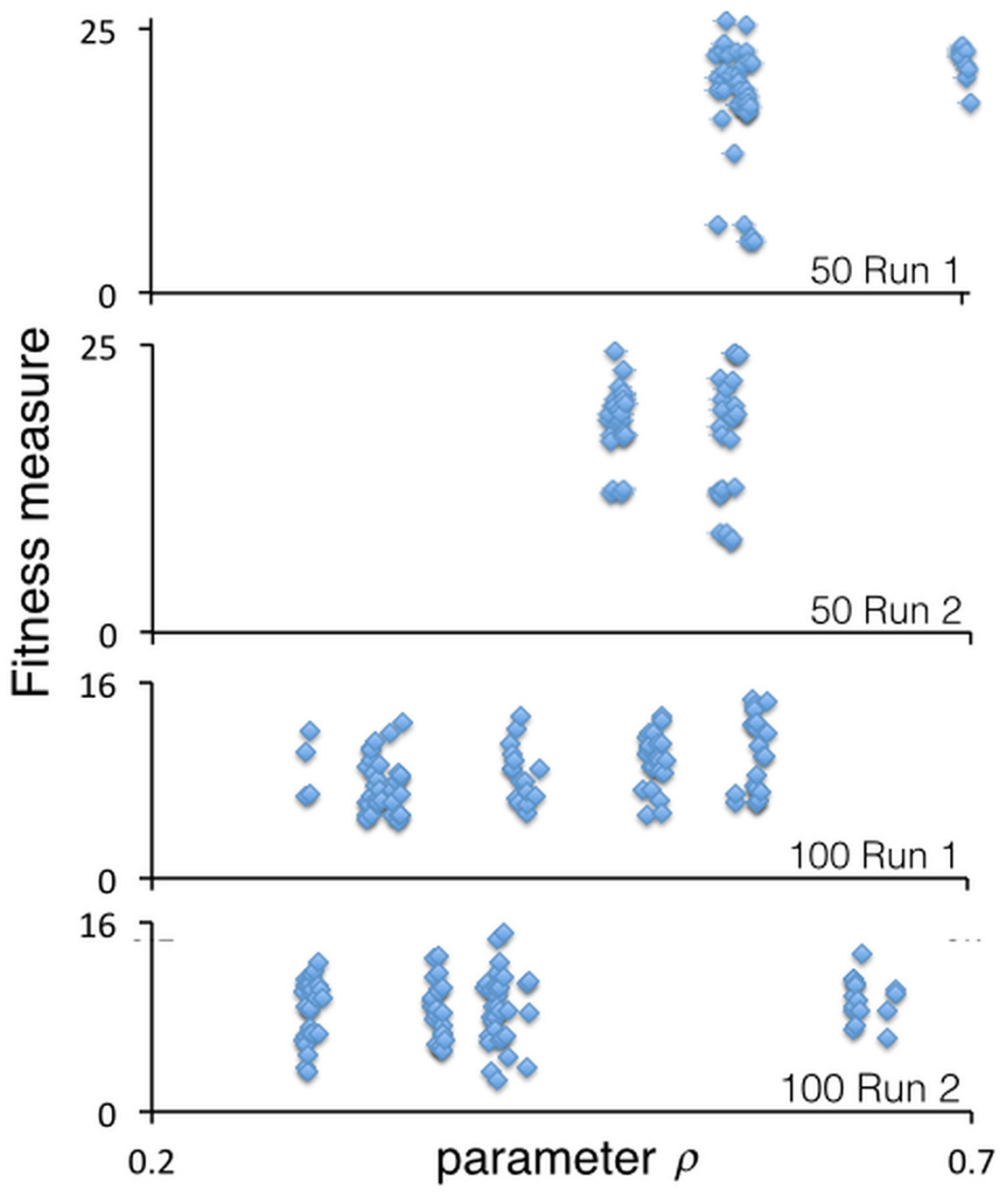
Evolved values of the movement-threshold parameter *ρ*. The four panels provide snapshots of the final values of *ρ* (*T* = 200), which range between just under 0.3 and just over 0.70 for the two 50-agent (two upper panels) and two 100-agent (two lower panels) runs, using the same baseline parameter values listed in Table 2.

In our third set of simulations, we increased the density of agents to 50 agents and in a fourth set of runs to 100 agents (i.e., densities of 0.125 and 0.25 individuals per patch respectively). Now the evolved outcomes after 200 generations become more interesting and structured than the low density 10-agent case. In two runs of 50 agents, the populations evolved into a 2–3 movement type polymorphism. For example, in Run 1 of the 50 agent case (top panel of Fig 4), we see a phenotype around *ρ* = 0.5 that actually appears to be two phenotypes with similar *ρ* values, and a phenotype with *ρ* = 0.7. In two runs of 100 agents, the populations evolved into around six different phenotypes in each case, (bottom two panels of Fig 4), though a couple of these phenotypes appear to have very similar *ρ* values.

**Fig 5:**
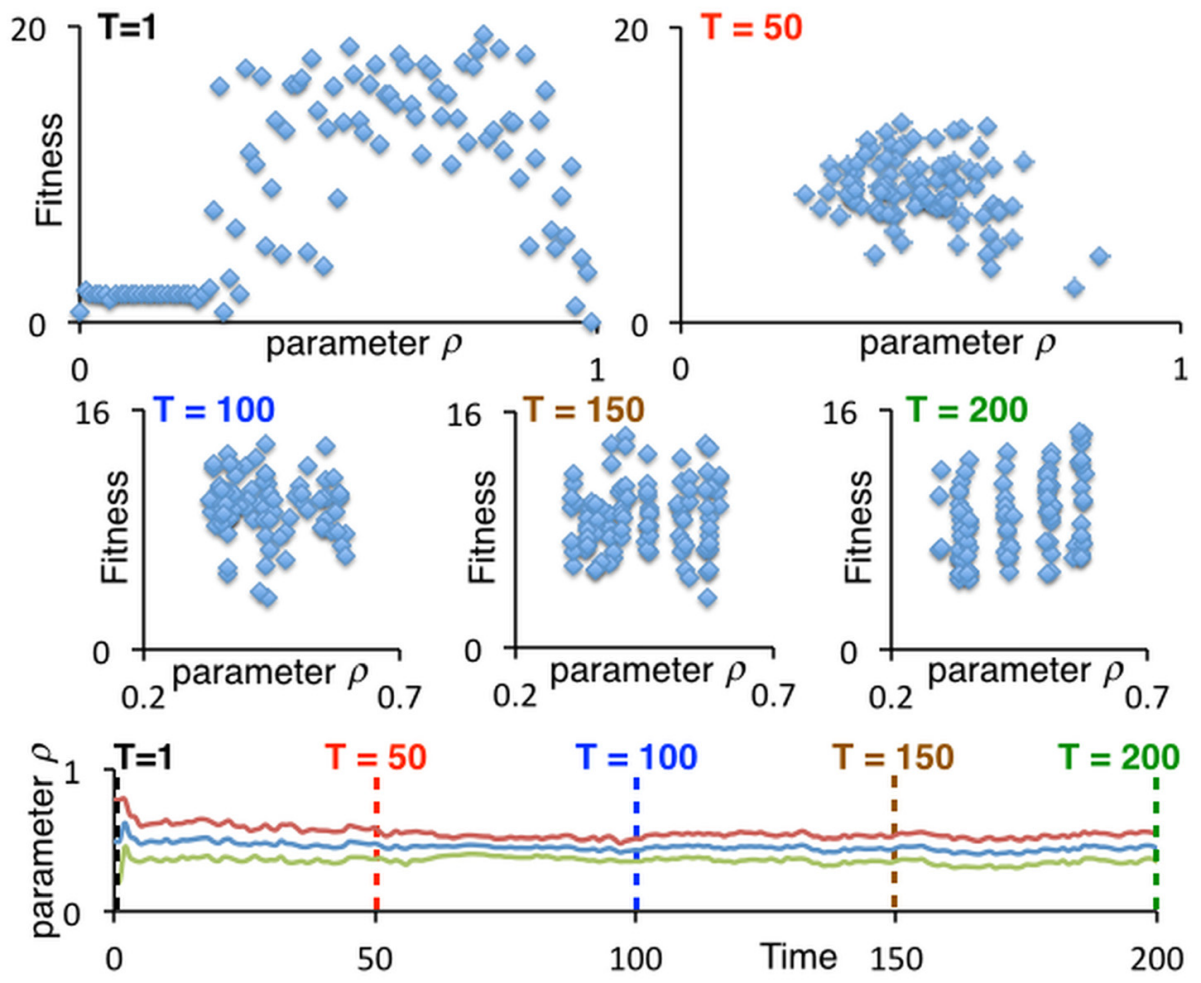
Evolution of the movement-threshold value *ρ*. The five scatter graphs (top two rows) represents snapshots over a particular 200-generation (*T*), 100-agent run of the parameter values *ρ* at times *T* = 1,50,100,150 and 200. The lower panel depicts the mean plus/minus the standard deviation of the values of *ρ* for the 100 agents over the interval 0 ≤ *T* ≤ 200.

Of course, evolution is an ongoing process, so we cannot be sure how the movement polymorphisms, illustrated in Fig 4, may continue to evolve after *T* = 200. To investigate this question, we took a detailed look at the evolutionary process over time by conducting a third run of the 100-agent case and taking snapshots of the evolving values of *ρ* at times *T* = 1, 50, 100, 150 and 200 (Fig 5). In this case, we see in the top left panel of Fig 5 that initially (*T* = 1) all values of *ρ* ∈ [0, 1] are evident (due to distributed selection of initial values of *ρ*), with low values of *ρ* (on the interval [0,0.25]) and values around 1 exhibiting low or even zero fitness. By the 50^*th*^ generation, the value of *ρ* for most agents lies between 0.3 and 0.8, with the upper threshold dropping below 0.7 by the 100^*th*^ generation. Beyond generation 100, a polymorphism of several movement types begins to emerge with all values of *ρ* roughly between 0.25, and 0.6. The heterogeneity in fitness across each morph (i.e., the vertical spread for each of the *ρ* values) is due primarily to chance events in which some individuals find themselves initially distributed over the landscape in denser areas than others. We note for the lowest panel in Fig 5, which depicts the average fitness plus/minus one standard deviation, in the value of *ρ* over the full 200-generation evolutionary epoch. We see that, initially, the high level of variance settles down and reaches a minimum around *T* = 70, when all the unfit individuals (i.e., those possessing low (< 0.25) and high (> 0.65) values of *ρ*) have been purged from the population. After that the variance begins to rise as the population organizes itself in polymorphism of around a half-dozen movement types on the interval *ρ* ∈ [0.25,0.65].

**Fig 6:**
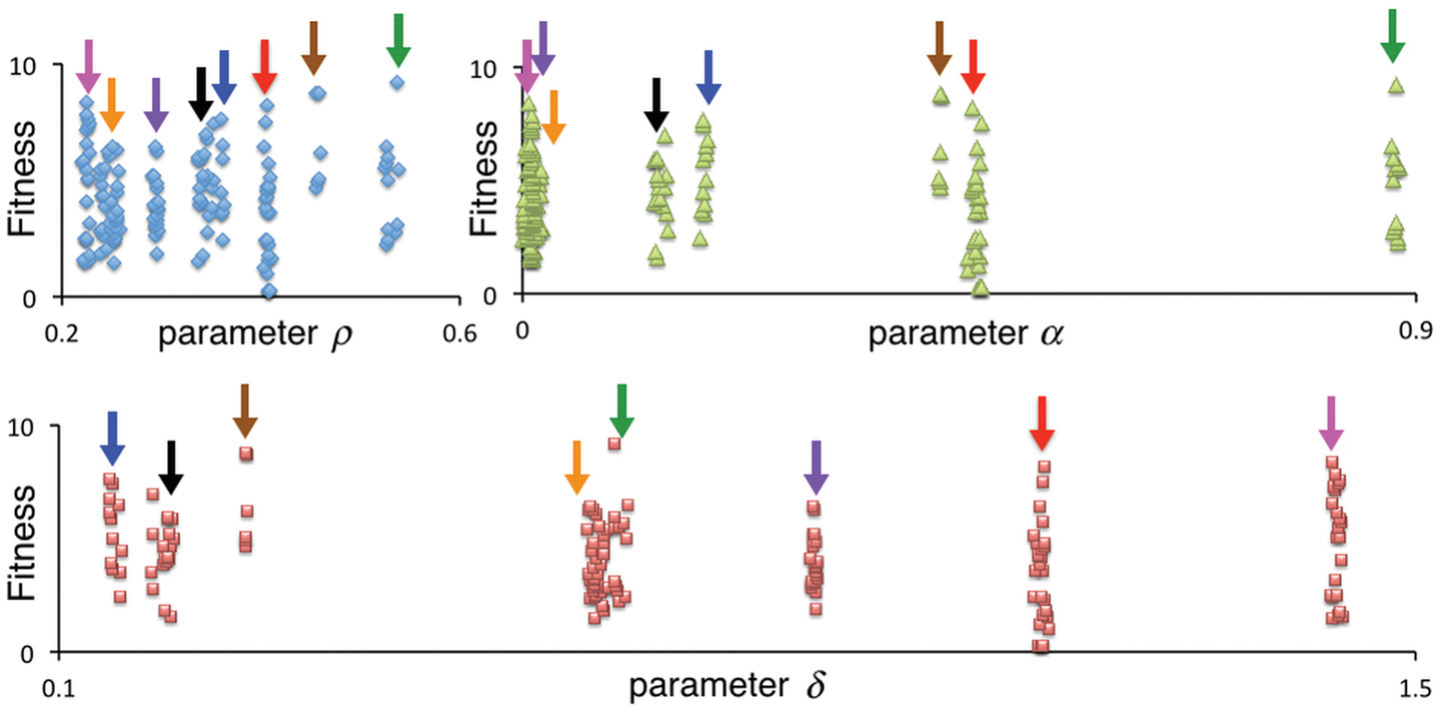
Evolved parameter values of different movement types in the 150-agent case. The final values (*T* = 200) of the three parameters *ρ* (blue diamonds), *α* (green triangles) and *δ* (red squares) are plotted for 150 individuals agents in the simulation, which we see have self-organized into eight different movement types (movement polymorphism), identified by the eight variously colored arrows: same colored arrows identify three parameter values that define each of the eight movement types.

The trend of increasing diversity in movement-type polymorphisms that evolves with increasing numbers of individuals is seen in a simulation with 150-agents. In Fig 6, we see that eight movement types emerge, as indicated by the 8 different colored arrows pointing to parameter triplets, (*α*, *δ*, *ρ*), where each color represents the parameter values associated with one type. These eight types are clearly indicated with regard to parameters *ρ* and *δ*, but three of the types that all have small *ρ* values (i.e., resist moving unless their current patch has relatively few resources) also have *α* values that are close to zero. This latter situation indicates an indifference to the resource and competitor values of second-tier neighbors (cells that are distance one removed for each individual’s immediate neighbors). For those individuals that are most likely to move out of their current cells (i.e., the green arrow, which corresponds to *ρ* ≈ 0.5), second-tier neighbors are relatively important (i.e., *α* = 0.9 for the green arrow type: see extreme left of upper left panel of Fig 6). We note that *δ* values can be both below and above 1, with the former weighting resources over competition and the latter associated with highly competition averse movement types. It is not surprising to see a high degree of polymorphism in the competition avoidance parameter *δ* since, if some individuals avoid competitors others can be more lax about avoiding competitors; in the extreme situation of all but one individual avoiding competitors, this individual can ignore the competition issue that is being taken care of by the others.

**Fig 7:**
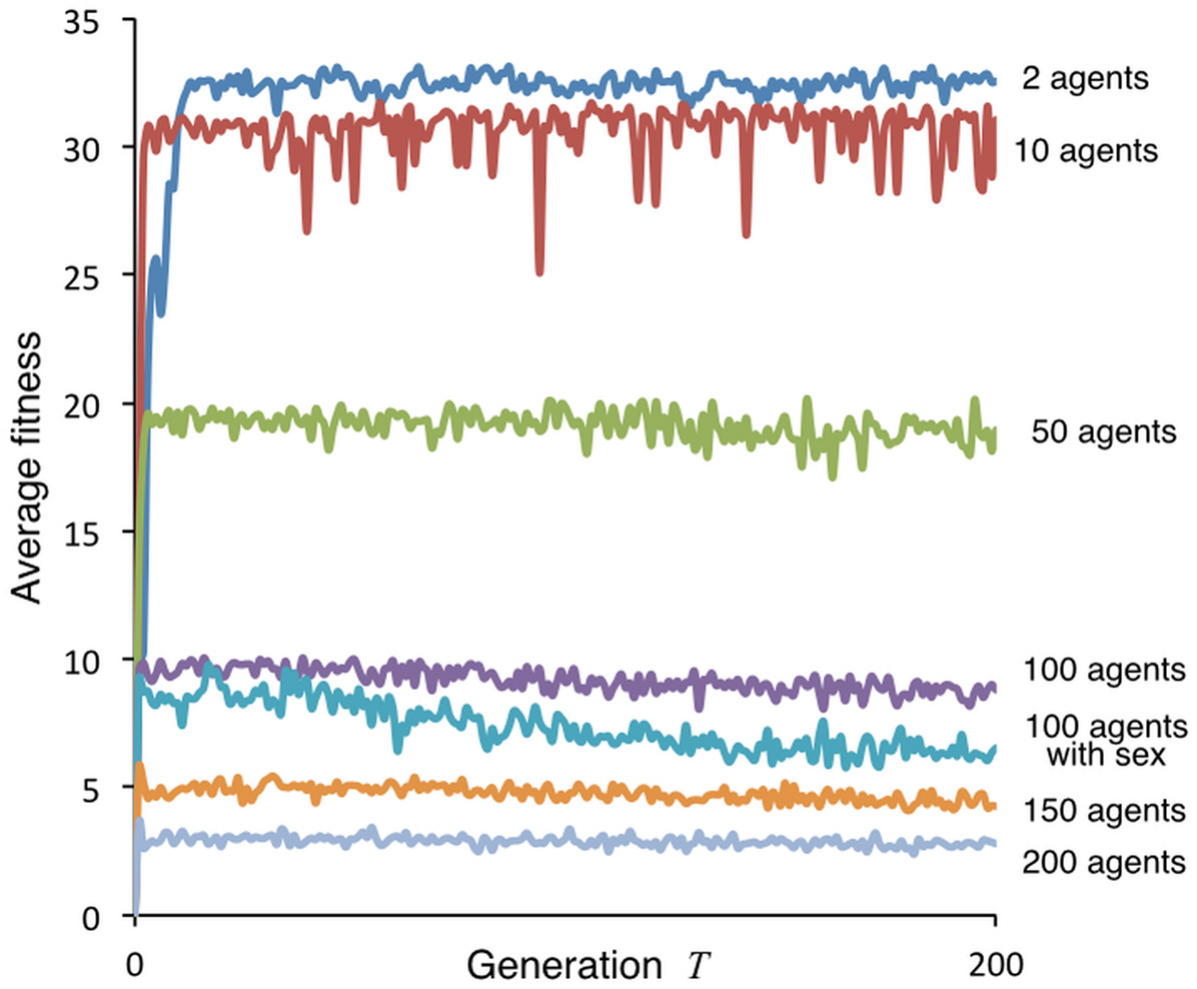
Time course of average fitness. (i.e., final biomass *B*_*a*_(*n*) among *N*_*A*_ agents). The average fitness is plotted over *T* = 1,…,200 generations for illustrative runs with the following number of clonal reproducing individuals, except for one sexually reproducing group, as indicated: 2 (blue), 10 (red), 50 (green), 100 (purple), 100 with sexual reproduction (turquoise), 150 (orange) and 200 (grey) agents.

Our final simulation in our exploratory series of clonally reproducing agent guilds was for the high density case of 200 agents. We then plotted the average 200-generation fitness trajectory obtain from this simulation along with representative runs for 2, 10, 50, 100, and 150-agent cases, as well as an exploratory run (see Figure in S3 Text) of 100 sexually reproducing hermaphroditic agents. In all cases, except the 2-agent case, the population rises within a few generations to reach the highest average fitness levels (Fig 7), with selection over time then having little effect on average fitness and, in some cases, even declining slightly (notice the slight downward trend in the 50, 100, and 150 agent cases in Fig 7). The reason for this slight downward trend is that as agents evolve to behave optimally so competition for resources stiffens to compensate for more effective movement decisions evolving at the individual level. Also losing diversity over time, as selection produces just a few movement types, leads to less efficient exploitation of resources from a population point of view—a phenomenon that is accentuated under sexual reproduction, as explored further in the study reported in the next section. The reason for the drop in biomass as the population evolved over time is that, from a population point of view, it is more efficient to have several different behavioral morphs exploiting a heterogeneous landscape, than just one type. Sexual reproduction, under the unrealistic assumption that we can ignore allelic structure when determining phenotype (i.e. by just assuming progeny are an average of their parents phenotype), results in a “fittest” behavioral type that may have locally maximized individual fitness in each generation, but has not maximized the collective biomass production rates of the evolved guild. This result suggests that it might be useful to undertake, as reported in the next section, a more detailed study of differences between the evolution of foraging guilds under clonal versus sexual reproduction, using a more realistic model of sexual reproduction that accounts for allelic structure in diploid organisms. This increase in realism leads to dramatically different conclusions regarding the emergent phenotypic structure of the evolved guild, thereby providing a cautionary tail against oversimplification.

## Clonal versus Sexual Reproduction

Our exploratory results support the hypothesis that the evolving collective biomass production efficiencies of clonally and sexually reproducing guilds of foragers, exploiting resources over patchy landscapes, are likely to differ considerably over time. To further address this hypothesis, we modified the sexual component of the simulation model discussed above to incorporate diploid genetics in which the behavioral phenotype of individuals (i.e. the expressed values of the trait parameters *α*, *δ* and *ρ*) is computed by averaging across two allelic values for each of the three phenotypic traits (i.e. codominent genes), where one allelic value for each trait is inherited from each parent following Mendelian rules for diploid genetic systems (random mating, random assortment of alleles, no linkage among traits). We did not assign a sex to agents, so the interpretation is that we are dealing with hermaphroditic (or monoecious) systems.

**Fig 8:**
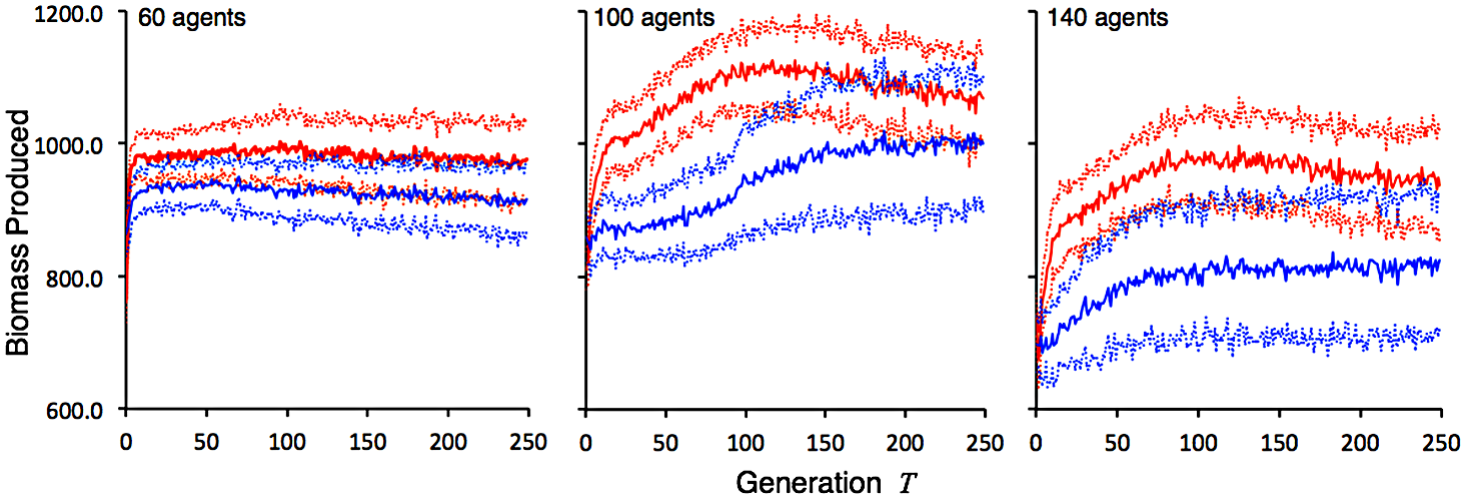
Evolving biomass production efficiency of guilds. Total biomass produced per generation (solid lines are averages over *n* runs, dotted lines are plus and minus one standard deviation) under clonal reproduction (red) and random mating (blue), by evolving guilds over 250 generations of, respectively from left to right, 60 (*n* = 50), 100 (*n* = 50), and 140 agent (*n* = 50) guilds of foragers. For a reference to these data see S4 Text.

We compared simulations (i.e. runs) using our hermaphroditic mating version of our model with the clonal version used in the exploratory phase for the cases of 60, 100 and 140 agents (note: we selected agent numbers divisible by four since each pair of parents produces 4 progeny). Output from initial simulations suggested that we should extend the 200 generation simulation interval used in our exploratory studies to 250 generations to obtain a better perspective on the long term behavior of our system. We repeated each simulation 50 times for the two reproduction modes (clonal and random) and the three agent densities (60, 100, 140). The total biomass produced (i.e. accumulated by each individual and summed across all individuals) each generation by each guild of agents for each of the different cases, was averaged over all the runs undertaken. The means and standard deviations over these fifty runs are plotted in Fig 8.

As expected, based on our exploratory results, the total amount of biomass produced by each guild of foragers in each generation was highly compensatory in terms of the number of agents, with both clonally and sexually reproducing guilds of 60 agents able to collectively produce almost as much each generation as guilds of 100 agents. As the number of agents increases, though, biomass production is hindered by competition, which we see in terms of a noticeable drop in total biomass produced by the 140-agent guild compareds with the corresponding (with regard to reproductive system) 100-agent guilds. Thus, guild exploitation is, in fact, overcompensatory. To formally test the significance of our results, we divided our simulations into two phases: an initial 125-generation “burn in” phase, and the subsequent 125-generation “approach to equilibrium” phase. From Fig 8 though, it appears in some cases that an equilibrium has been reached, while in others (e.g. the 60 agent random and 100 agent clonal cases) the amount produced over generations 126 to 250 appears to be declining slightly over time. The results of this analysis are tabulated in Table 4, where we see clonally evolving guilds of foragers produce more biomass in total over both phases of the evolutionary process than randomly mating guilds of foragers. We also expected greater levels of variation associated with the randomly mating guilds, but this was only true (i.e. highly significant) for the 100-agent and 140-agent cases. In the 60-agent case, the reverse was true, with randomly mating guilds showing significantly less variation than clonally mating guilds. The major difference between the 60-agent versus the 100 and 140-agent cases is that in the latter two interspecific competition becomes a much more important factor, as seen from the compensatory behavior of the total biomass produced by the guild in the 60-agent, 100-agent, and 140-agent comparisons (Fig 8).

**Table 4:** Foraging guild biomass extraction efficiency: total produced over the two labeled 125-generation periods.

Period	Clonal		Random mating		Two-sided *T*-test*	
	Biomass	$\sqrt{{\text{Var}}_{1}}$	Biomass	$\sqrt{{\text{Var}}_{2}}$	Significance	Var_1_ ≠ Var_2_
60 Agents					*n* = 50	
1–125	122829	1769	116409	1067	*p* < 0.0001	*p* ≈ 0.0006
126–250	122286	41272	115044	2644	*p* < 0.000	*p* ≈ 0.0022
100 Agents					*n* = 50	
1–125	131857	1739	112606	3440	*p* < 0.0001	*p* < 0.0001
126–250	136228	2958	124074	10136	*p* < 0.0001	*p* < 0.0001
140 Agents					*n* = 50	
1–125	116017	1764	96895	7645	*p* < 0.0001	*p* < 0.0001
126–250	119859	3129	101711	10854	*p* < 0.0001	*p* < 0.0001

* Tests run at URL: http://www.quantitativeskills.com/sisa/statistics/t-test.htm

**Fig 9:**
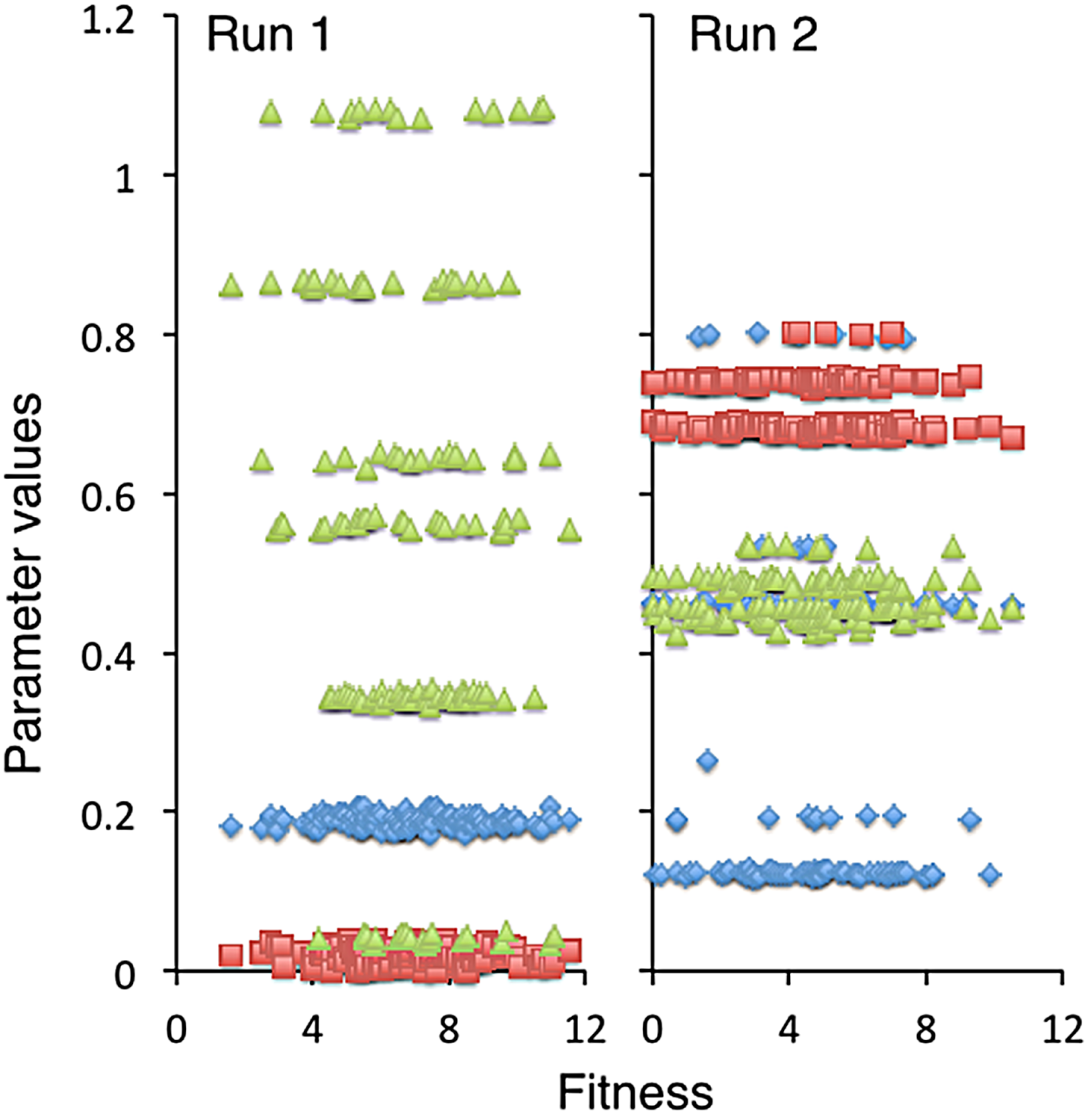
Evolved values of parameters under random mating. Parameter phenotype values for each of the 140 agents in the final generation (*T* = 250) of the first two of 50 simulations are plotted as a function of their fitnesses (ranging from 0 to 12). The evolved *neighbor-discount* parameter *α* (green triangles), *competition-tradeoff* parameter *δ* (red squares), and *movement-threshold* parameter *ρ* (blue diamonds), across the 140 agents differ in the two runs and show different degrees of dispersion, but reflect both heterogeneous and homogeneous phenotypes: viz. in Run 1 we see six *α* phenotypes that arise from the emergence of three alleles, as discussed in Table 5.

To understand in more detail the genetic structure of the foraging guilds that evolve under random mating, we plotted the parameter values that emerged in the first two runs of our 50-run, 140-agent simulations (Fig 9), and tabulated (Table 5) the allelic aspects of the first of these runs (left panel in Fig 9). For comparative purposes, we also tabulated (Table 5) the phenotypic trait values for the first of our 50-run, 140-agent clonal reproduction simulations. In Table 5, we see for the clonal case that seven foraging phenotypes evolved from a random mating configuration during the 250-generation evolutionary epoch. The neighbor-discount parameter *α* exhibits the widest range of variation (around 0.06 to 1.52, noting that the values in Table 5 are the evolved values multiplied by 10^3^), while the competition-tradeoff *δ* and movement-threshold parameter *ρ* exhibit considerably less variation (around -0.03 to 0.04, and 0.12 to 0.21, respectively). For the random mating case, the phenotypic trait values can be unpacked according to underlying diploid genotypes. In Run 1 (left panel of Fig 9), as with the first clonal run, we see that the neighbor-discount parameter *α* exhibits the widest range of variation compared with competition-tradeoff *δ* and movement-threshold *ρ* parameter values. The results of Run 2 (right panel of Fig 9) are different, though, in that the movement-threshold parameter *ρ* now exhibits the widest range of variation, but noticeable variation in the other two parameters is still evident. In Run 1, the underlying genetic basis of the the six neighbor-discount *α* phenotypes are three distinct alleles (also the values of the homozygote phenotypes because of codominant averaging), as indicated in the Table 5, that have the approximate (because of mutational variation) values 0.045, 0.645 and 1.03 respectively, with the three possible heterozygote phenotypes being the intermediate to these values. In Run 2, we see a number of phenotypes that have evolved, with the underlying genetic structure, as evident in the right panel of Fig 9 that the phenotypes are based on two alleles (one close to 0.7 and one to 0.8) for the competition-tradeoff trait, three alleles (all three relatively close in value, lying between 0.40 and 0.55) for the neighbor-discount trait, and three alleles (more spread out in value between 0.1 and 0.8) for the movement-threshold trait. We note in Table 5 that the 140-agents are in Hardy-Weinberg equilibrium, which is expected since this equilibrium arises in the progeny due to random mating and random segregation of alleles. Selection only acts at the end of each generation, when individuals are sorted according to how much biomass they have produced. We hypothesize that evolved random mating guilds are less efficient than evolved clonally reproducing guilds, as evident in Fig 9, because heterozygotes are constrained to be intermediate to homozygotes in the random mating case, while clonal phenotype strategy guilds are not under such constraints, allowing them to be optimally spaced by the evolutionary process. Such an analysis, however, is beyond the scope of this paper, particularly since other factors in our model (such as hard versus soft selection) need to be modified to make our simulations more realistic, as discussed in the next section.

**Table 5:** Foraging guild phenotypes at the end of 140 agent exemplar runs.

Clonal reproduction		Parameter phenotype values			
Type	Number	*α* × 10^3^	*δ* × 10^3^	*ρ* × 10^3^	Biomass
1	52	67 ± 3	23 ± 2	192 ± 5	6.3 ± 2.2
2	12	209 ± 2	37 ± 1	206 ± 2	6.8 ± 1.2
3	18	240 ± 1	9 ± 5	204 ± 3	7.0 ± 2.0
4	22	758 ± 2	7 ± 2	156 ± 5	7.3 ± 2.5
5	4	808 ± 1	42 ± 1	180 ± 3	7.1 ± 1.6
6	20	1191 ± 2	26 ± 3	157 ± 2	6.8 ± 2.7
7	12	1520 ± 01	−31 ± 1	124 ± 2	5.8 ± 3.7
Total	140				6.6 ± 2.4
Random mating		Parameter phenotype values			
Type	Obs (Exp^‡^)	*α* × 10^3^	*δ* × 10^3^	*ρ* × 10^3^	Biomass
*Genotypes* ^†^					
*α*_1_ *α*_1_ *δδρρ*	22 (20.8)	45 ± 5	9 ± 13	188 ± 9	7.3 ± 1.8
*α*_1_ *α*_2_ *δδρρ*	38 (37.0)	345 ± 5	18 ± 14	189 ± 9	7.1 ± 1.5
*α*_2_ *α*_2_ *δδρρ*	18 (16.5)	645 ± 4	18 ± 9	188 ± 8	7.2 ± 2.1
*α*_2_ *α*_3_ *δδρρ*	22 (26.1)	864 ± 3	19 ± 11	187 ± 7	5.9 ± 2.4
*α*_3_ *α*_3_ *δδρρ*	14 (10.3)	1031 ± 4	18 ± 15	188 ± 7	7.05 ± 2.5
*α*_1_ *α*_3_ *δδρρ*	26 (29.3)	562 ± 5	19 ± 8	1928 ± 8	6.8 ± 2.4
Total	140				6.9 ± 2.1

^†^ Although more than one allele is evident for *δ* and *ρ* that variation is low (cf. left panel in Fig 9): so we only sort on *α*

^‡^ Hardy-Weinberg equilibrium theory: differences between observed and expected not significant

## Discussion and Conclusion

Analyses of hierarchical, multi-scale ecological systems have long been of concern to ecologists [40]. It is only in the last decade, however, that it has become reasonable to simulate such systems in terms of access to adequate, inexpensive computational power. Methodologies for evaluating the output of complex systems have been discussed for nearly two decades [41–43], but our ability to communicate the structure of complex ecological models remains hampered by the lack of facility to visually understand the structure of modules at different hierarchical levels (but see Fig 2). Frameworks have been proposed for modeling complex systems (e.g. complex adaptive landscape (CAL) framework for modeling complex adaptive systems occurring on heterogeneously structured landscapes [44]), particularly across multiples scales when spatial structure is included (cf. [45] in the context of epidemiological systems). Increasingly, scientists are making their code available to others to build upon; but this code typically does not help in visualizing the underlying model structure. For example, a recent, agent-based modeling study to assess the impact of landscape fragmentation on diseases transmission [46], included details of the model written in Java; but, as such, the code is only accessible to experienced Java programmers as a basis for building more elaborate models.

In the model developed here, we have endeavored to make the underlying architecture of our code as transparent as possible, by providing figures of the Nova model components at each hierarchical level of the four nested computational clocks employed in our analysis (individuals in cells, intragenerational, evolutionary epoch, repeated simulations to obtain statistical data). This level of visualization of model design is a departure from our previous agent-based studies, written in MATLAB [47]. These earlier MATLAB models were used to address questions regarding the evolution of specialization among individuals exploiting a mix of resource types (the leitmotif being insects selecting different plant types on which to lay their eggs—see [20]), and provide insight into the emergence of Batesian mimicry among vulnerable “resource-individuals” trying to mimic the aposematic signals of individuals protected from exploitation [32]. In the first of these earlier studies, we modeled a mix of generalists and specialist exploiters, in terms of how these exploiters use several versus only one type of plant. In these systems, a guild of exploiter types typically emerged, much as in our results reported here. These new results reveal the following characteristics that we could not a priori infer or anticipate: i) the number of foraging strategy types emerging as a result of clonal reproduction varied and increased with the density of agents per unit cell; ii) the foraging strategy types that constituted an emerging polymorphism under clonal reproduction varied with runs, but showed some repeatable and some variable characteristics (e.g. at the very low densities of 2 to 10 agents, the movement-threshold parameter *ρ* evolved each time to a value in the range 0.7 to 0.8, though the other two parameters could vary greatly, as in the neighbor-discount parameter *α* evolving to 0 or to 1); iii.) under random mating, foraging strategy polymorphisms evolve and create several distinct alleles per trait.

The existence of polymorphisms is a necessary but not sufficient precursor for the occurrence of sympatric speciation ([31, 48, 49]). Holmgren and colleagues studied the process of speciation among individuals specializing in exploiting particular resource types by, first, replacing clonal reproduction with sexual reproduction and then studying the divergence of specialists in the context of assortative mating within particular exploiter types [32]. In the study reported here, we found that it was not necessary to have assortative mating for the maintenance of strategy polymorphisms or, more surprisingly, even more than one resource type. Such polymorphisms are a precursor for the occurrence of sympatric speciation on patchy landscapes: i.e. a precursor for heteropatric speciation, as defined by Getz and Kaitala [50]. The inclusion of assortative mating in future studies, facilitated say through the inclusion of metapopulation structures or genes for mate selection linked to genes for strategy type (as discussed by Norrstrom et al. in the context of habitat selection [31]), is likely to enhance the emergence and maintenance of diverse foraging strategy guilds and, ultimately, sympatric speciation.

Computational methods have been used to address questions relating to the behavior of populations in the context of complex systems theory, with the emergence of collective behavior of biological populations being a case in point. In the past, such questions have typically been of a rather general or theoretical nature [51–53], as are the questions addressed here regarding the emergence of polymorphisms of foraging strategy types and how such polymorphisms may be affected by sexual reproduction. This work could easily be extended by, say, including a more sophisticated treatment of patch heterogeneity in terms of growth and recovery rates of resources within patches, the effects of patch aggregation and landscape structure on movement behavior [54], and the effects of assortative or disassortative mating structures on the emergence of behavioral (forging strategy in our case) polymorphisms (e.g. see [31, 55]). Also, the assumption of hard selection (i.e. a fixed number of progeny are produced each generation) could be replaced with a soft selection assumption (i.e. the fecundity of individuals depends on the amount of biomass they produce each generation) to make the models ecologically more realistic [56]. Within such an extended framework, a host of interesting evolutionary questions can be investigated as they may relate to the emergence or existence of dominant/recessive allelic relationships, recognition systems, sex-ratio dynamics, sexual selection and so on. Since the options appear to be innumerable, beyond purely theoretical questions the development and application of models is best executed with specific systems in mind. Cases in point are computationally intensive models used to investigate the carrion finding strategies of griffon vultures [57] or predict the movement of banana stem weevils in the banana plantations [58].

Although it has been easier in the past to use agent-based models to address general rather than specific-systems questions, because the latter generally have “many more moving parts” and involve landscape specific data handled by GIS software, we have no doubt that the field of systems-specific computational biology will burgeon, as the capabilities of software grow to easily code and handle such models. We have demonstrated here that the *Nova* platform is moving us towards these desired software capabilities. As a result of reducing the considerable burden of coding complex agent-based models and casting them within a genetic algorithmic framework that now is easily implemented using the *Nova* platform, in the future we should be able to study evolutionary process with much greater ease. We anticipate that such studies will lead to a plethora of now insights, with the study reported here providing a taste of things to come. In particular, we provide incontrovertible evidence here that a population of randomly mating foragers, exploiting a single, randomly-distributed set of resource patches can (and may well be likely to) evolve into and be maintained as a polymorphic foraging-strategy guild.

## Supporting Information

S1 Table (PDF)

S1 Text (PDF)

S2 Text (PDF)

S3 Text (PDF)

S4 Text (PDF)
