# Supplementary material for: Panmictic and Clonal Evolution on a Single Patchy Resource Produces Polymorphic Foraging Guilds
Source: PLoS One. 2015 Aug 14;10(8):e0133732. doi: 10.1371/journal.pone.0133732 (PMC4537111; doi:10.1371/journal.pone.0133732)
Supplement: S1 Table — (PDF) [file pone.0133732.s001.pdf]

S1 Table. Model Notation

Time

T: current generation time
G: maximum generation time
t: intra-generation time
n: maximum intra-generation time

Agent associated designators

A_a: agent a
N_A: number of agents
$E_a(t)$: local environment of agent a at time t
$B_a(t)$: biomass of agent a at time t
$L_a(t)$: location of agent a at time t

Agent movement

$M_(P_a)^a$ specifies if and where to agent a will move at time t,
as determined by a vector of parameters P_a
α: competition-tradeoff parameter (parameter a in Nova code)
δ: neighbor-discount parameter (parameter b in Nova code)
ρ: movement-threshold parameter (parameter w in Nova code)

Agent consumption

u: maximum resource extraction rate
h: the extraction efficiency parameter
q: the competition parameter
κ: biomass conversion rate parameter
c: metabolic loss rate parameter
r: resource intrinsic growth rate
g: reservoir parameter
s: saturation parameter

Cells

C_{ij}: cell at row i and column j in the array
C_k: cell k, where $k = 1, , n_i n_j$ is a 1-D renaming of the matrix 2-D array
$R_k(t)$: resource value of cell k at time t
$N_k(t)$: number of agents remaining in cell k after movement at time t
J_k^M average number of agents per cell in the Moore neighborhood of cell k
$F_k(t)$ resource extraction by an agent in cell k at time t
