# Supplementary material for: Panmictic and Clonal Evolution on a Single Patchy Resource Produces Polymorphic Foraging Guilds
Source: PLoS One. 2015 Aug 14;10(8):e0133732. doi: 10.1371/journal.pone.0133732 (PMC4537111; doi:10.1371/journal.pone.0133732)
Supplement: S1 Text — (PDF) [file pone.0133732.s002.pdf]

S1 Text. The Nova platform

As mentioned in the main text, the Nova platform is used to build modularized capsules that interact with their environments through input and output interfaces or channels ([10]; cf. Fig. 1 in main text). A particularly simple capsule is setting up a growth equation, either for a population or for an individual (agent). Further, the model can take the form of either an iterative mapping that generates a sequence of points or a numerical integration over a specified time interval (modeled by the agent capsules depicted in Fig. 1 of the main text). These capsules can be used as elements in more complex models (as long as there is no circularity), communicating with other elements using input and output channels that are graphically represented by pins, with connections specified using pull-down menus (see Figure S2 Text).

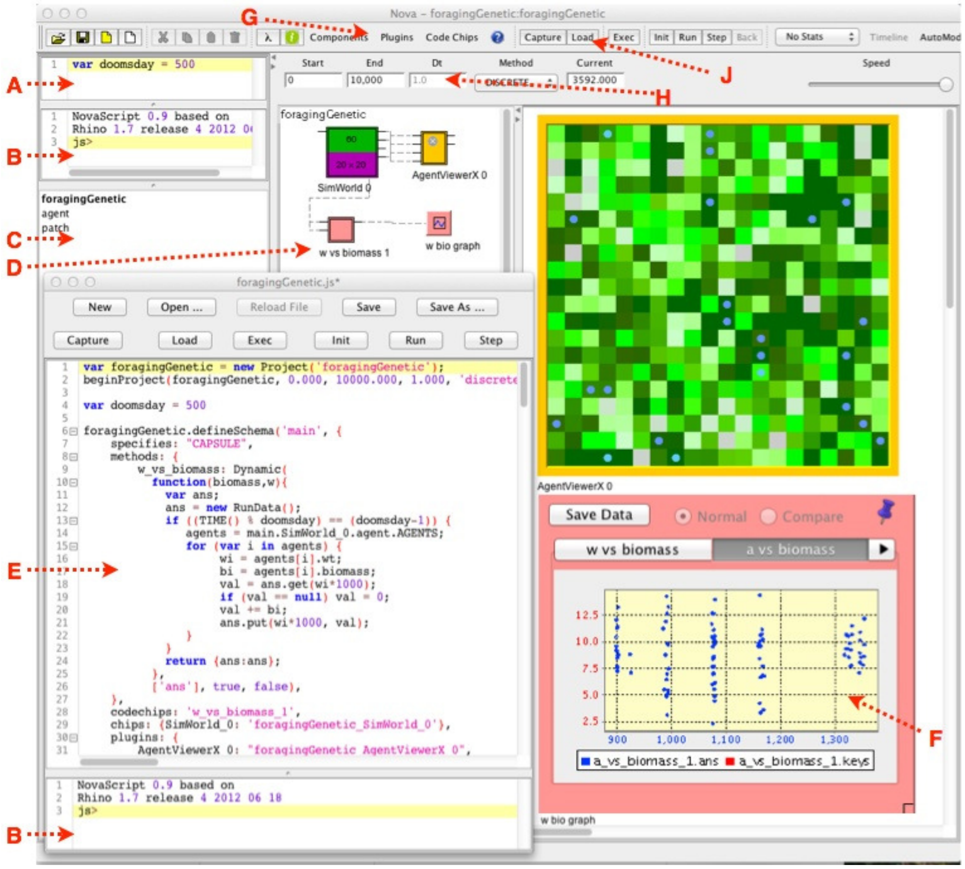

Figure. The Nova GUI contains the following windows: A. runtime specification panel; B. NovaScript control console; C: hierarchical list of model capsules; D: canvas where modules are constructed using icons; E. optional floating window with captured NovaScript code; F. dashboard that displays controls, graphs, tables and visualization plugins; G: buttons to activate floating **Component**, **Plugins**, and **Code Chips** pallets; H. simulation specification windows; J. buttons to **Capture**, **Load**, and **Execute** the code, and to initialize, **Run**, or **Step** through the simulation.

If a capsule computes a change in the state of an individual, then a set of capsules can be managed using one of Nova's aggregators, such as an *agent-vector aggregator* or a *simworld aggregator* that links sets of agents with sets of cells topologically organized as an array (rectangular or hexagonal), as depicted in Fig. 1 of the main text, or

organized into a network specified using a non-negative connectance matrix: if the ij^{th} entry is positive then the ith and j^{th} cells are connected, either bidirectionally when the ji^{th} entry is also positive or directionally if the latter is zero. Further, when required, the magnitude of these entries represent the strengths of connections. Instances of capsules, called chips, may appear in other capsules, where the functionality of a chip is that of the model it encapsulates. Capsules exist as individual entities that can be imported and exported, so that they may be reused when needed in other modules or models.

Computational instructions in the form of equation, or code in "code chips", are specified in dialogue boxes that are opened when right clicking on flow icons (equations) or code chips (computational instructions). These instructions follow JavaScript syntax and protocols. When all graphical elements have been appropriately hooked together, clicking the **Capture** and **Load** buttons on the Nova graphical user interface (see Figure S2 Text) results in the creation of a runable NovaScript program. NovaScript is an extension of the JavaScript language that executes once the **Exec** button is clicked.
