# Supplementary material for: Panmictic and Clonal Evolution on a Single Patchy Resource Produces Polymorphic Foraging Guilds
Source: PLoS One. 2015 Aug 14;10(8):e0133732. doi: 10.1371/journal.pone.0133732 (PMC4537111; doi:10.1371/journal.pone.0133732)
Supplement: S2 Text — (PDF) [file pone.0133732.s003.pdf]

S2 Text. Nova and movement on cellular arrays

In the Nova platform, agents can move over a landscape specified by: i) a rectangular or hexagonal cellular array of any dimension (the only constraint are hardware and software resources to run the program), ii) points in the Cartesian plane (or volume for three dimensional movement), iii) a network of patches with topology specified by a connectance matrix, or iv) a combined Euclidean-space/cellular-array, where sets of Euclidean points are mapped to particular cells in the array.

The advantage of hexagonal over rectangular grids is that hexagonal grids have only one type of neighborhood (list of cells that are neighbors to a given cell), while rectangular grids have two (Figure S3 Text). As a consequence, calculating how many cell-to-cell moves are required to get from one cell to another on a rectangular array, depends on whether diagonal moves are allowed (i.e., Moore neighborhood moves defining eight directions) or forbidden (i.e., von Neumann neighborhood moves defining four directions). On a hexagonal array, six movement directions are possible, each defining six symmetric movement cones (Figure S3 Text).

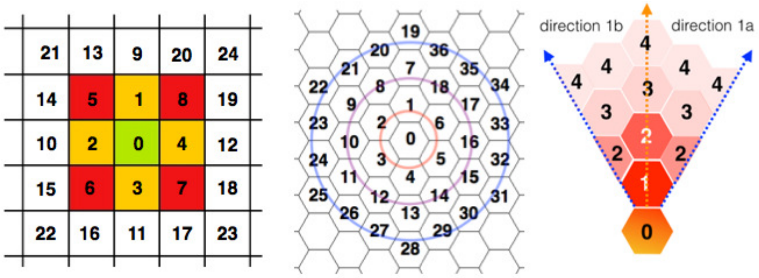

Figure. Cells in rectangular arrays have two types of neighborhoods, von Neumann (left panel, orange squares) and Moore (left panel, orange and red squares). The minimum number of cell-to-neighboring-cell steps to get from cell 0, say, to cell 13 is three for von Neumann neighborhoods but only two for Moore neighborhoods because diagonal moves are now permitted. Hexagonal arrays have only one type of neighborhood with distances, based on cell-to-cell moves, easily demarcated (e.g. distances of one to three moves from cell 0 are represented in the middle panel by red, purple and blue circles respectively). Cones of directions with respect to cell 0 are also easily determined in terms of the six directions: 1 north, 2 northwest, 3 southwest, 4 south, 5 southeast and 6 northeast (middle panel). Each direction defines a cone of cells (e.g. right panel depicts the north directional cone), with possible further splits into left and right half of these symmetrically identified cones. Note these cones contain both full and, on their boundaries, bisected cells (right panel). Also hexagonal arrays can be oriented so that the directions are: west, southwest, southeast, east, northeast, northwest.

On a hexagonal array, an individual can use directional cones to calculate resource availability in each direction, discounting the value of the resources in particular cells based on distance (i.e., the number of moves needed to reach that cell) from the individual's current cell location. Resources in half-cells within directional cones (i.e., the left and right edge cells at even distances from the focal cell), as depicted in the left panel in Figure S3 Text, can additionally be discounted by a factor of two. Directional cones can also be used to assess potential levels of agonistic interactions, or danger from predators, by counting relevant individuals of different types in these directional cones, also discounted by distance and half cell inclusions. For example, if R_i represents the resources available in cell i in the hexagonal array depicted in the central panel in Figure S3 Text, then a weighted measure of the resources available in direction 6, within

a distance of three steps from cell 0, may be calculated, using suitably chosen weighting parameters $w_1 > 0$, $w_2 > 0$ and $w_3 > 0$, as

$$R_6^{\mathbf{w}} = w_1 R_6 + w_2 \left(\frac{R_{16}}{2} + R_{17} + \frac{R_{18}}{2} \right) + w_3 \left(R_{33} + R_{34} + R_{35} \right) \tag{2}$$

Such calculations may then be used by an individual in cell 0, containing resources R_0, to decide whether and where to move, as illustrated by the movement rule in S2 Text.

Hexagonal Movement (resources only)

1. For each agent A_a, $a = 1, ..., N_A$, at time t, identify its current location $L_a(t)$.
2. Then, agent-by-agent, labeling $L_a(t)$ as cell 0 for the agent in question, calculate $R_i^{\mathbf{w}}(t)$, $i = 1, ..., 6$, as specified in Eqn. 2 and its corresponding forms for $i = 1, ..., 5$.
3. Use the values $R_i^{\mathbf{w}}(t)$, $i = 1, ..., 6$, along with the value $R_0(t)$ and a threshold value ρ to determine if and where to move according to a rule, such as:

   $$\begin{array}{llll} \textit{If} \ \dfrac{R_i^{\mathbf{w}}}{R_0} < \rho \ \ i = 1, \ldots, 6 & \textit{Then} & \textbf{Stay} \\ & \textit{Else} & \textbf{Move to cell } i, \\ & & \text{where } \ i = \text{argmax}\{R_j^{\mathbf{w}} | j = 1, \ldots, 6\} \end{array}$$

   In this case the movement parameter vector is $\mathbf{p}_a = (w_1, w_2, w_3, \rho)'$ ($'$ denotes vector transpose), where all values in $\mathbf{p}_a$ are agent specific.

Note that in S2 Text, the environmental inputs are

$$\mathbf{E}_a(t) = \{R_0(t), R_1^{\mathbf{w}}(t), \ldots, R_6^{\mathbf{w}}(t) | \text{location of cell 0 is } L_a(t)\}$$

and the movement designator $M_{\mathbf{p}_a}^a$ is determined purely in terms of the vector $\mathbf{E}_a(t)$.

As evident in Figure S3 Text, on rectangular arrays either four or eight directions arise, depending on whether diagonal moves are allowed. If they are, then cells 9-24 in the left panel of Figure S3 Text, are two-steps away from cell 0, even though the corner cells have centers that are $\sqrt{2}$ further away than the centers of the due north, south, east, and west cells from cell 0. This could have some relevance if individual movement is on a Cartesian plane, where energy is lost in proportion to the underlying Euclidean distance. This problem can be mitigated when using Moore movement neighborhoods in the context of an underlying Cartesian plane, if we adopt an approach along the lines outlined in Box 4 (see Main text).
