# Supplementary material for: Panmictic and Clonal Evolution on a Single Patchy Resource Produces Polymorphic Foraging Guilds
Source: PLoS One. 2015 Aug 14;10(8):e0133732. doi: 10.1371/journal.pone.0133732 (PMC4537111; doi:10.1371/journal.pone.0133732)
Supplement: S3 Text — (PDF) [file pone.0133732.s004.pdf]

S3 Text. Exploratory simulations in a sexually reproducing guild

As an exploratory investigation of the impacts of sexual reproduction on an evolving guild of agents, we carried out simulations of hermaphroditically reproducing individuals for the 100-agent case. Given the exploratory nature of this simulation, we kept the genetics simple by ignoring ploidy in assuming that the parameter phenotypes of the progeny (i.e. the three trait parameter values) were the averages of its two parents, with the possibility of mutational perturbations to these parameter values from one generation to the next. In our main study, however, we consider the more complicated diploid genetic systems of trait inheritance. In the simulations undertaken in this exploratory study, we simply allowed individuals in the fittest half of each generation (i.e., in this case 50 individuals) to choose mates at random among themselves, with each of the 25 parental couples so arising producing four progeny, thereby restoring the cohort to 100 individuals at the start of each intragenerational cycle. Not unexpectedly, as seen in Figure S4 Text, under panmictic (random) mating, hermaphroditic reproduction leads to the emergence of a single movement morph. This 'fixed' morph type, however, differs from run to run due to stochastic factors. In the first of these runs, as seen in Fig. 7 (main text), the average biomass started out around 9.26 and then dropped steadily over 200 generations to 6.53, as the population evolved from a random assortment of movement types to a single movement type. In the second run, the average started out at 9.81 biomass and dropped to 6.38 after 200 generations. Repeated runs gave similar results, though the actual combination of evolved parameter values differed from run to run.

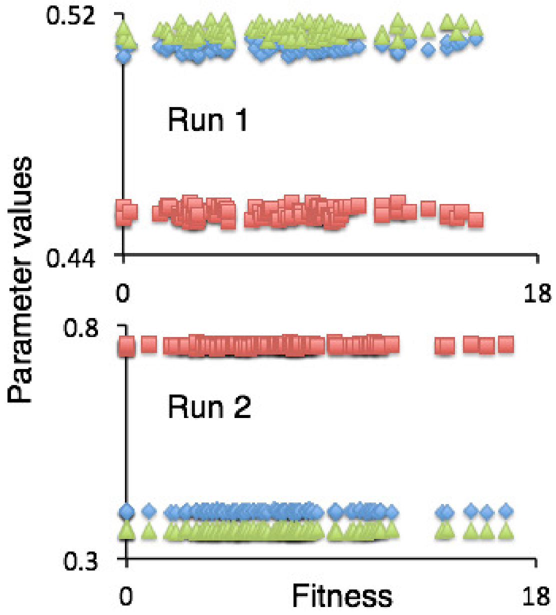

Figure. Evolved values of parameters for 100 sexually-reproducing agents, when ploidy structure is ignored. The top and bottom panels are the result from two different 100-agent runs using the baseline data (Table 2), but now sexual reproduction and random mating is included, as discussed in the text. Parameter values for each of the 100 agents in the final generation ($T = 200$) are plotted as a function of their fitnesses (ranging from 0 and 18). The evolved *competition-tradeoff* parameter α (green triangles), *neighbor-discount* parameter δ (red squares), and *movement-threshold*

parameter ρ (blue diamonds) differ across the 100 agents. The two runs yield different movement morphs (note the vertical axis scales are different for the two runs). Thus, unlike the 100-agent clonal reproduction case, with ρ parameter values plotted in Fig. 4 in the main text, only one movement type now evolves in each run. This situation strongly contrasts with the polymorphic structure that is produced when diploid, co-dominant inheritance of alleles are incorporated into the mating process.
