# Supplementary material for: Panmictic and Clonal Evolution on a Single Patchy Resource Produces Polymorphic Foraging Guilds
Source: PLoS One. 2015 Aug 14;10(8):e0133732. doi: 10.1371/journal.pone.0133732 (PMC4537111; doi:10.1371/journal.pone.0133732)
Supplement: S4 Text — (PDF) [file pone.0133732.s005.pdf]

S4 Text. Location of data and running the model

A Nova Online version of the model can be found at http://novaol.oberlin.edu/genetic. The username is: PLOSONE; the password is: PLOSONE. Also, data plotted in Figure 8 are located at: http://dx.doi.org/10.6084/m9.figshare.1477998
